# The Mask of Delirium: Uncovering a Presumed Cardioembolic Stroke Through Comprehensive Clinical Evaluation

**DOI:** 10.7759/cureus.116336

**Published:** 2026-09-16

**Authors:** Prakash Velmurugan, Sarath Kumar Mohan Kumar, Sadaf Shahzad, Ruhee Moryani Chand, Vikram Paranjyothi

**Affiliations:** 1 Geriatric Medicine, Medway NHS Foundation Trust, Gillingham, GBR; 2 Gastroenterology, Medway NHS Foundation Trust, Gillingham, GBR; 3 General Internal Medicine, Medway NHS Foundation Trust, Gillingham, GBR; 4 Geriatric Medicine, Medway Maritime Hospital, Gillingham, GBR

**Keywords:** acute ischaemic stroke, delirium, language assessment, older adults, presumed cardioembolic stroke, wernicke aphasia

## Abstract

Delirium and aphasia exhibit overlapping clinical features, complicating the diagnosis of acute neurological disturbances in older adults. Both conditions impair communication and orientation, leading to incoherence; however, they arise from distinct pathophysiological mechanisms and require different management strategies. This case concerns an 83-year-old woman with a 48-hour history of acute-onset confusion and abnormal speech. Magnetic resonance imaging (MRI) revealed an acute infarct in the inferior division of the left middle cerebral artery (MCA) territory, with cardioembolism considered the likely cause. Initial assessment attributed her fluent but semantically meaningless speech, inattention, and fluctuating responsiveness to hyperactive or mixed delirium superimposed on probable pre-existing cognitive impairment. New-onset atrial fibrillation (AF) with a controlled ventricular rate was identified, along with elevated high-sensitivity cardiac troponin I concentrations (1026.3 and 1060.5 ng/L) and N-terminal pro-B-type natriuretic peptide (7451 pg/mL) in the context of severe ventricular dysfunction.

Treatment for possible acute coronary syndrome was initiated but discontinued after cardiology review. Persistent and stereotyped language abnormalities prompted systematic neurological re-evaluation, which identified a Wernicke-type aphasia profile. Computed tomography (CT) of the head demonstrated a left parietotemporal infarct. Given the 48-hour clinical presentation, MRI was performed to further characterise the lesion and revealed left temporal diffusion-weighted imaging (DWI) hyperintensity with corresponding apparent diffusion coefficient (ADC) hypointensity, confirming acute restricted diffusion in the left temporal lobe. The MRI abnormality corresponded anatomically with the left temporal component of the CT finding, although movement artefact prevented precise lesion co-registration. Echocardiography demonstrated severe biventricular dysfunction with a left ventricular ejection fraction of 15% to 20%. This case underscores the clinical risks of misattributing a structured language disorder to delirium and highlights the importance of focused neurological assessment, including systematic language evaluation, in all older adults presenting with acute confusion.

## Introduction

Delirium is a neuropsychiatric syndrome defined by an acute-onset disturbance in attention and awareness that develops over a short period and fluctuates throughout the day [1]. A multicenter audit across UK hospitals reported a delirium prevalence of 16.3% among hospitalised older adults [2]. Predisposing factors include advanced age, dementia, sensory impairment, and functional dependence, while precipitating factors encompass infection, metabolic disturbance, polypharmacy, and acute medical illness [3]. Clinical manifestations are heterogeneous, ranging from hyperactive agitation and verbal incoherence to hypoactive withdrawal, and are associated with increased mortality, prolonged hospitalisation, and accelerated cognitive decline [3,4].

In contrast, stroke is defined as a focal neurological deficit of sudden onset caused by interruption of cerebral blood supply, either through vessel occlusion (ischemic stroke) or haemorrhage [5]. Among the cardinal signs of stroke, speech and language disturbances, including aphasia, dysarthria, and dysphasia, are particularly prominent and prognostically significant [6]. Aphasia, an acquired disorder of language production and/or comprehension due to focal brain injury, complicates approximately 30% of ischemic strokes, with risk increasing in older patients [7]. Wernicke’s (receptive) aphasia, resulting from infarction of the posterior superior temporal gyrus supplied by the inferior division of the left MCA, is characterised by fluent, prosodically normal but semantically empty speech, frequent neologisms and paraphasias, severely impaired comprehension, and anosognosia [8].

A meta-analysis reported a pooled incidence of Wernicke’s aphasia of 13% among post-stroke aphasia cases [9]. Despite distinct pathophysiological origins, delirium and aphasia share features that often complicate differentiation in cases of apparent disorientation [3,8]. The presence of AF at stroke onset and during the acute phase was confirmed by electrocardiogram in 24.6% of patients with ischemic stroke [10]. Elevated troponin is a key factor in the co-occurrence of ischemic stroke and atrial fibrillation, as well as in the development of adverse outcomes [11]. Such cardiac findings may redirect clinical attention toward a cardiac aetiology, potentially delaying recognition of underlying stroke. This case is presented to illustrate the structured clinical approach that corrected the diagnosis and to discuss the overlapping features of delirium and Wernicke-type aphasia in acute cardioembolic stroke.

## Case presentation

An 83-year-old woman was brought to the emergency department by her daughter, who found her at home in a state of acute confusion. Over the preceding 48 hours, the patient became unable to recognise familiar faces, ceased producing coherent speech, and was unable to recall names or respond appropriately to questions. The daughter described the speech as wrong words, all muddled, and observed that her mother appeared completely unaware of her communication difficulties. There was no history of head injury, chest pain, syncope, fever, dysuria, or witnessed seizure.

The patient's medical history included hypertension, knee osteoarthritis, and a previous mastectomy for breast carcinoma in 2004. She had stopped drinking alcohol six months earlier. She had been living independently and mobilising without aids and had recently been referred to a memory clinic for intermittent cognitive difficulties, although no formal diagnosis of dementia had been established. On arrival, the patient was haemodynamically stable, afebrile (temperature 36.8°C), and maintaining her airway independently. The Glasgow Coma Scale score was 14/15, with eye opening to voice, a confused verbal response, and normal motor responses. The initial clinical impression was hyperactive or mixed delirium superimposed on probable underlying dementia. The admitting team observed agitation, continuous speech, and apparent disorientation to time, place, and person. Notably, the specific linguistic characteristics of her speech were not formally evaluated at this stage: verbal output, though fluent in rate and prosody, was semantically empty and consisted entirely of neologistic jargon. The patient appeared unaware of her communication failure and laughed incongruously at intervals. No focal motor deficit was identified.

Investigations

Cardiovascular examination revealed bilateral pitting oedema to the knees and an irregular pulse. The initial full blood count demonstrated thrombocytosis without anaemia or leucocytosis (Table 1).

**Table 1:** Full blood count. Results are shown for admission day one and subsequent inpatient sampling days; the final column gives the local laboratory reference interval. g/L: grams per litre; L/L: litres per litre; pg: picograms; fL: femtolitres; %: percent; ×10¹²/L and ×10⁹/L, cell counts per litre.

Parameters	Day 1	Day 2	Day 4	Day 15	Reference interval
Haemoglobin	133	132	138	147	115–165 g/L
Haematocrit	0.42	0.41	0.43	0.46	0.37–0.47 L/L
Mean corpuscular haemoglobin (MCH)	26.3	26.4	26.2	26.0	27–32 pg
Mean corpuscular haemoglobin concentration (MCHC)	320	323	322	329	320–360 g/L
Mean corpuscular volume (MCV)	82.4	81.8	81.4	80.5	80–100 fL
Red cell distribution width (RDW)	15.4	15.2	15.7	15.4	11.5–14.5%
Red blood cell count (RBC)	5.07	4.99	5.26	5.65	3.8–5.8 ×10¹²/L
Platelet count	434	447	492	536	140–400 ×10⁹/L
White blood cell count (WBC)	7.6	8.8	10.7	6.4	3.6–11.0 ×10⁹/L

C-reactive protein was mildly elevated (Table 2).

**Table 2:** Inflammatory marker. Results are shown for admission day one and subsequent inpatient sampling days. mg/L: milligrams per litre; <: less than. The reference interval is that which is reported by the local laboratory.

Parameter	Day 1	Day 2	Day 4	Day 15	Reference interval
C-reactive protein (CRP)	18.8	21.5	17.0	2.6	<5 mg/L

Renal function and electrolyte testing showed creatinine within the reference interval and initial hypokalaemia, with serum potassium concentrations of 3.2-3.3 mmol/L, which normalised on subsequent testing (Table 3).

**Table 3:** Renal function and electrolytes. Acute kidney injury stage 0 means that no laboratory acute kidney injury alert was generated. mL/min/1.73 m²: millilitres per minute per 1.73 square metres of body surface area; µmol/L: micromoles per litre; mmol/L, millimoles per litre; >, greater than. Adjusted calcium is the albumin-corrected calcium concentration. Reference intervals are those reported by the local laboratory.

Parameters	Day 1	Day 2	Day 4	Day 15	Reference interval
Estimated glomerular filtration rate (eGFR)	72	83	77	69	>90 mL/min/1.73m²
Creatinine	68	60	64	70	45–84 µmol/L
Acute kidney injury (AKI) stage	0	0	0	0	0
Sodium	139	136	137	139	135–146 mmol/L
Potassium	3.3	3.2	3.6	4.0	3.5–5.3 mmol/L
Adjusted calcium	2.37	2.39	2.43	2.51	2.1–2.6 mmol/L
Magnesium	0.79	0.72	0.68	0.87	0.70–1.00 mmol/L
Phosphate	1.09	0.99	0.89	1.19	0.8–1.5 mmol/L

Liver function testing demonstrated a mild transient rise in bilirubin, while alkaline phosphatase, alanine transaminase, and albumin remained within their respective reference intervals (Table 4).

**Table 4:** Liver function tests. Results are shown for admission day one and subsequent inpatient sampling days. µmol/L: micromoles per litre; IU/L: international units per litre; g/L: grams per litre. Reference intervals are those reported by the local laboratory.

Parameters	Day 1	Day 2	Day 4	Day 15	Reference interval
Total bilirubin	27	36	30	19	0–21 µmol/L
Alkaline phosphatase	85	84	87	91	30–130 IU/L
Alanine transaminase	16	16	22	11	10–40 IU/L
Albumin	38	36	38	39	35–50 g/L

Thyroid function tests were normal (Table 5).

**Table 5:** Thyroid function test. The results were obtained on day 2 of admission. pmol/L: picomoles per litre; mIU/L: milli-international units per litre. Reference intervals are those reported by the local laboratory.

Parameters	Day 2	Reference interval
Free thyroxine (free T4)	13.1	12–22 pmol/L
Thyroid-stimulating hormone (TSH)	3.72	0.27–4.5 mIU/L

Cardiac biomarkers demonstrated elevated high-sensitivity cardiac troponin I concentrations of 1026.3 and 1060.5 ng/L and an N-terminal pro-B-type natriuretic peptide (NT-proBNP) concentration of 7451 pg/mL (Table 6).

NT-proBNP was not repeated because, in accordance with local trust guidance, it was used for the initial assessment of heart failure rather than serial monitoring. Admission electrocardiography demonstrated new-onset atrial fibrillation with a controlled ventricular rate (Figure 1). The troponin elevation and ECG findings initially prompted treatment for possible non-ST-elevation myocardial infarction through an acute coronary syndrome (ACS) pathway. Cardiology later considered ACS unlikely and stopped ACS-focused treatment. Potassium replacement and fluid-balance management continued.

**Table 6:** Cardiac biomarkers. The first and second samples refer to serial samples collected on admission. ng/L: nanograms per litre; pg/mL, picograms per millilitre. Reference intervals are those reported by the local laboratory.

Parameters	Day 1 (first sample)	Day 1 (second sample)	Reference interval
High-sensitivity cardiac troponin I (ng/L)	1026.3	1060.5	0.0–17.5 ng/L
N-terminal pro-B-type natriuretic peptide (NT-proBNP) (pg/mL)	7451		0–399 pg/mL

**Figure 1:**
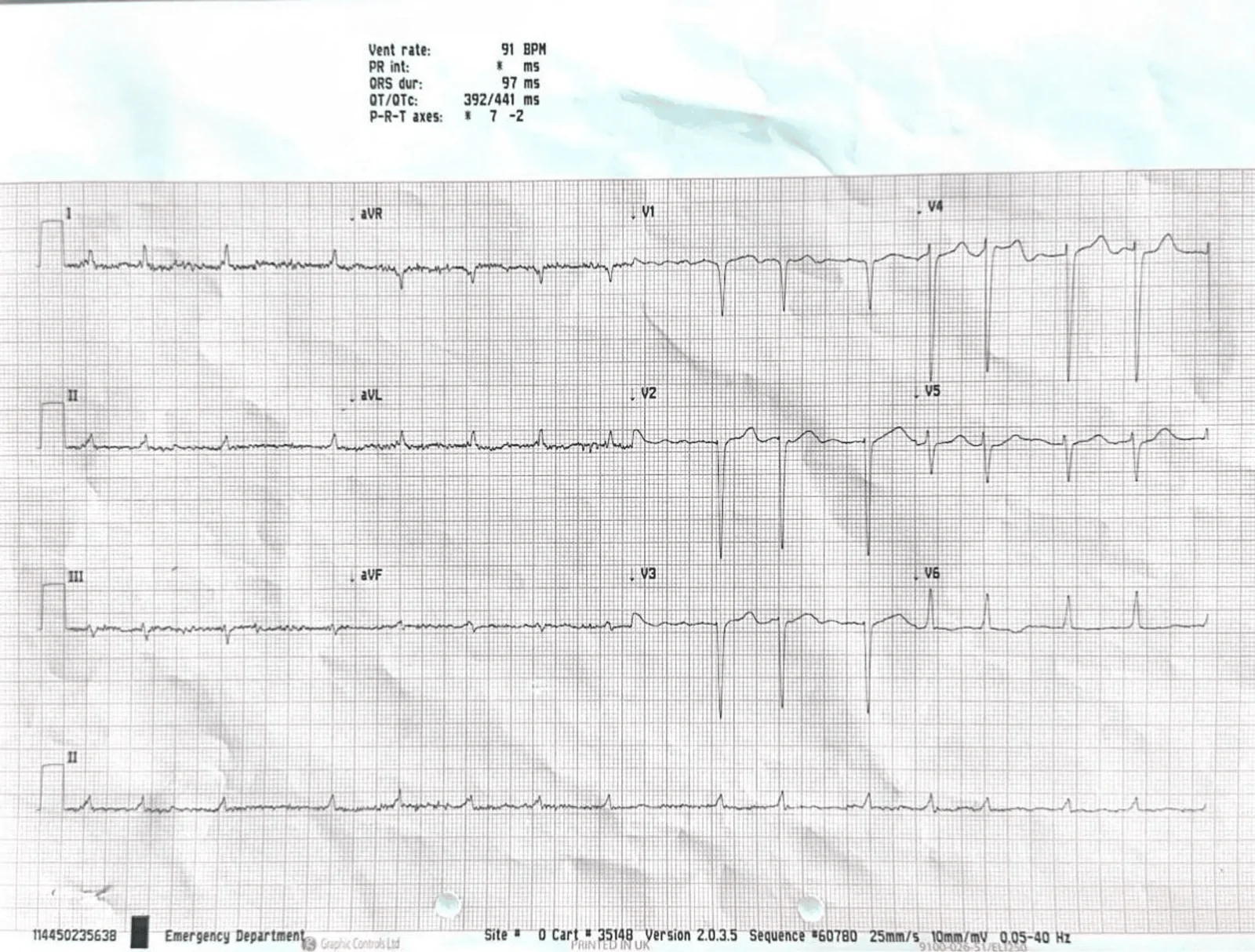
Twelve-lead ECG on admission demonstrating new-onset atrial fibrillation with controlled ventricular rate. The tracing demonstrates atrial fibrillation with a controlled ventricular response. ECG: electrocardiogram; AF, atrial fibrillation.

Non-contrast computed tomography (CT) of the brain demonstrated a left parietotemporal infarct without haemorrhagic transformation, bilateral small-vessel ischaemic changes, hippocampal atrophy (medial temporal lobe score 2 [12]), and no acute haemorrhage (Figure 2).

**Figure 2:**
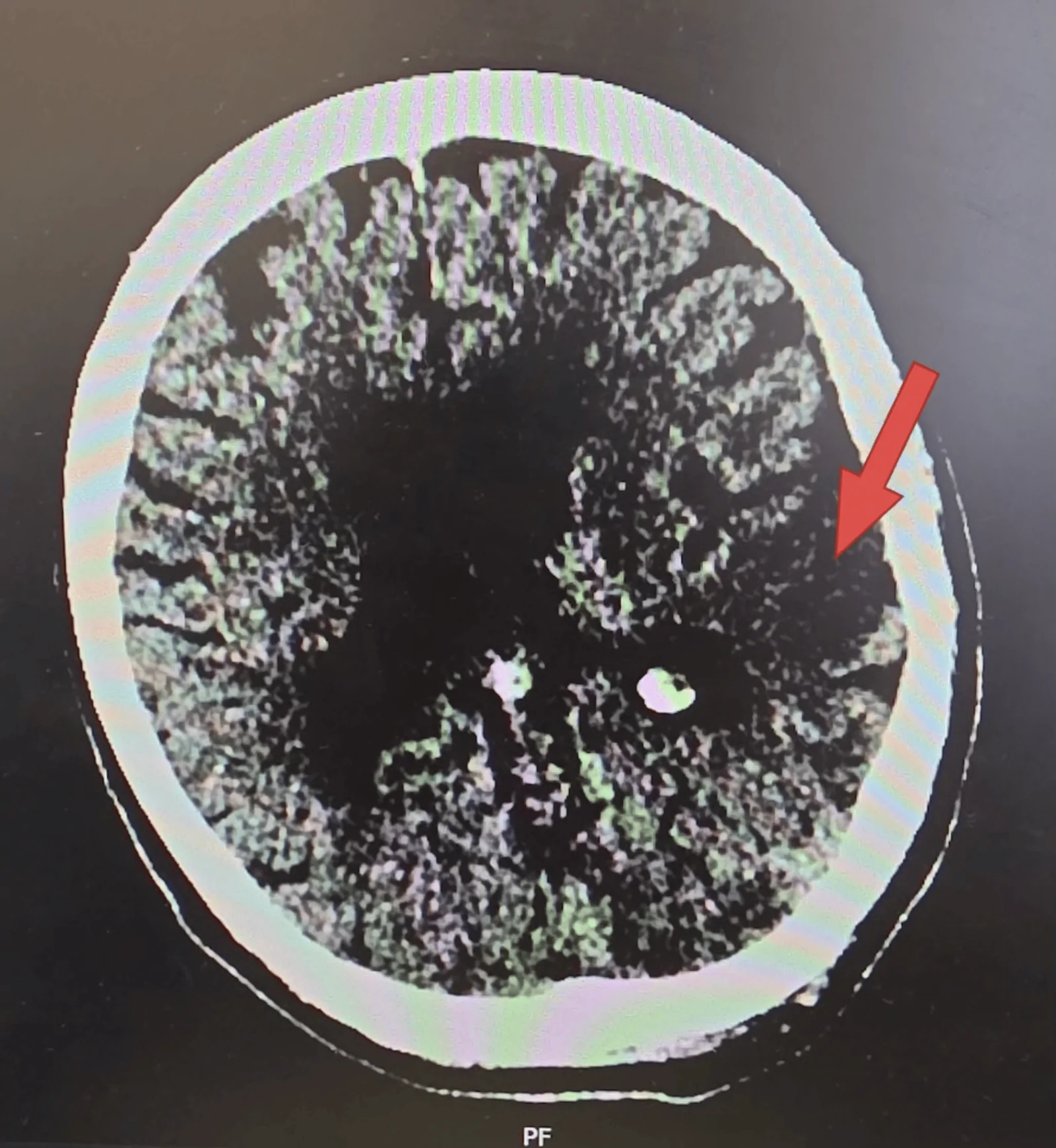
Axial non-contrast CT image of the brain. The red arrow indicates the left parietotemporal infarct. No haemorrhagic transformation was identified.

Chest radiography was unremarkable. Over the subsequent days, the patient was managed for delirium with possible underlying dementia using standard non-pharmacological interventions, including reorientation, exposure to familiar voices, a low-stimulus environment, avoidance of unnecessary sedation, and regular review of reversible precipitants [15]. Despite these measures, a clinical pattern inconsistent with delirium emerged: verbal behaviour remained stereotyped across assessments rather than fluctuating, as is typical in delirium. The patient consistently produced fluent, prosodically intact speech without meaningful content, did not reliably respond to yes/no questions, and could not follow single-step commands or point to named objects. These findings prompted neurological and speech-language evaluation.

Structured language assessment identified neologistic jargon with no meaningful content, complete failure in object-naming (zero out of five), inability to follow single-step instructions across verbal, written, and modelled modalities, and inability to repeat, count, read, or write meaningfully. This constellation of findings was recognised as severe receptive and expressive impairment with a Wernicke-type profile, rather than delirium alone. Stereotyped fluent output, profound comprehension failure, and lack of awareness of the deficit indicated a posterior dominant-hemisphere cortical lesion rather than a global attentional disturbance [3,8]. Given the 48-hour deterioration and absence of a prior focal language deficit, magnetic resonance imaging (MRI) was performed to further characterise the lesion.

Transthoracic echocardiography revealed severe biventricular systolic dysfunction with a left ventricular ejection fraction of 15% to 20%, consistent with previously undiagnosed advanced heart failure with reduced ejection fraction (HFrEF). MRI, although degraded by movement artefact, demonstrated diffusion-weighted imaging (DWI) hyperintensity with corresponding apparent diffusion coefficient (ADC) hypointensity in the left temporal lobe, confirming acute restricted diffusion. The MRI abnormality was anatomically compatible with the left temporal component of the CT finding, although movement artefact precluded exact lesion co-registration.

The dominant left temporal or parietotemporal location, Wernicke-type phenotype, and absence of persistent major motor weakness are anatomically consistent with an inferior-division left middle cerebral artery (MCA) distribution [13]. No CT angiography, MR angiography, arterial occlusion, or MCA thrombus was documented; thus, the territory designation remains anatomical rather than angiographically confirmed. New-onset atrial fibrillation (AF) made cardioembolism plausible, but the mechanism is considered presumed rather than proven. The fluid-attenuated inversion recovery (FLAIR) abnormality is shown in Figure 3, DWI high signal in the left temporal region in Figure 4, and the corresponding low ADC signal in Figure 5.

**Figure 3:**
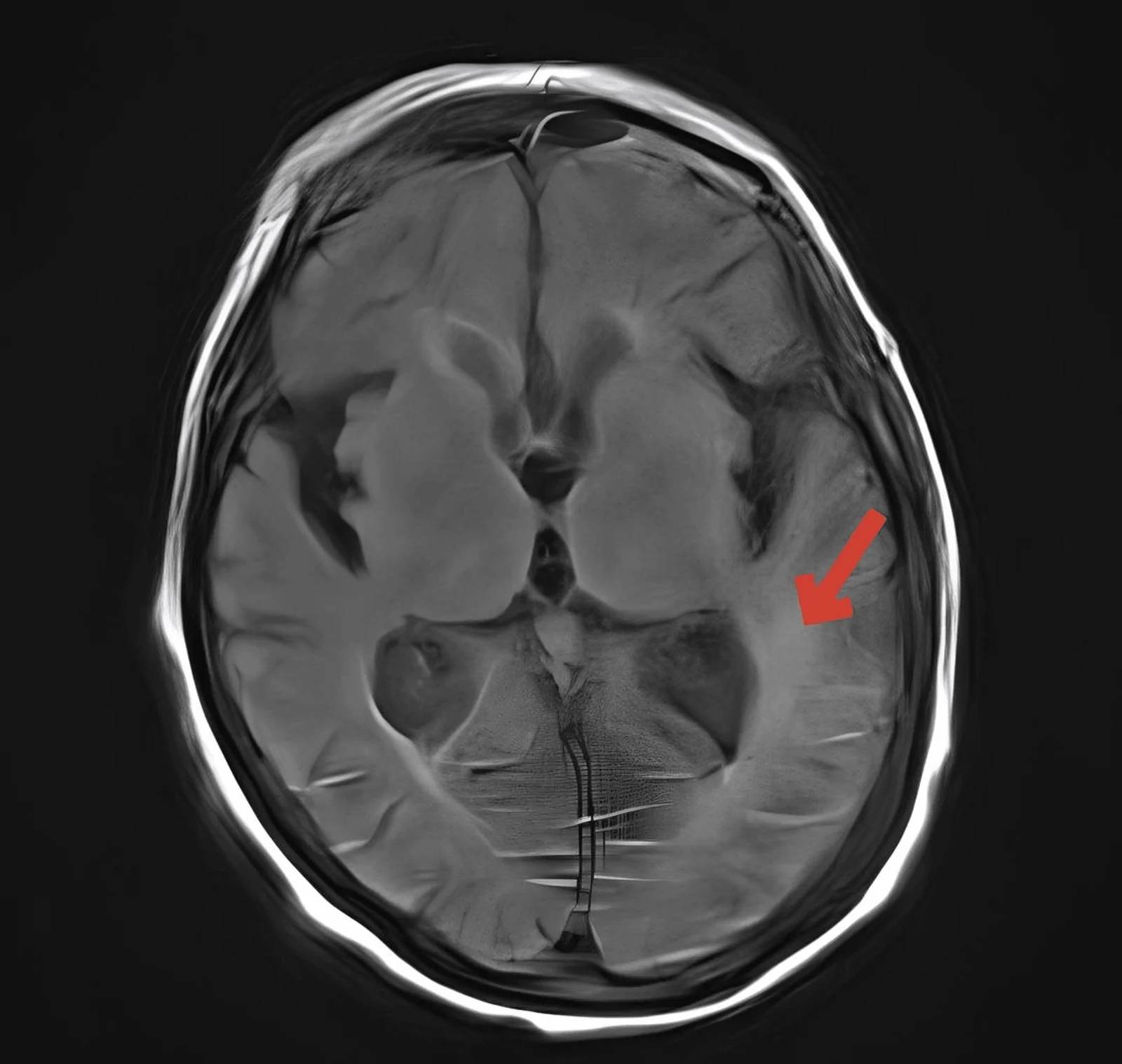
Axial fluid-attenuated inversion recovery MRI of the brain. The image shows hyperintense signal in the left temporoparietal region. FLAIR, fluid-attenuated inversion recovery; MRI, magnetic resonance imaging. FLAIR hyperintensity alone does not signify acute infarct.

**Figure 4:**
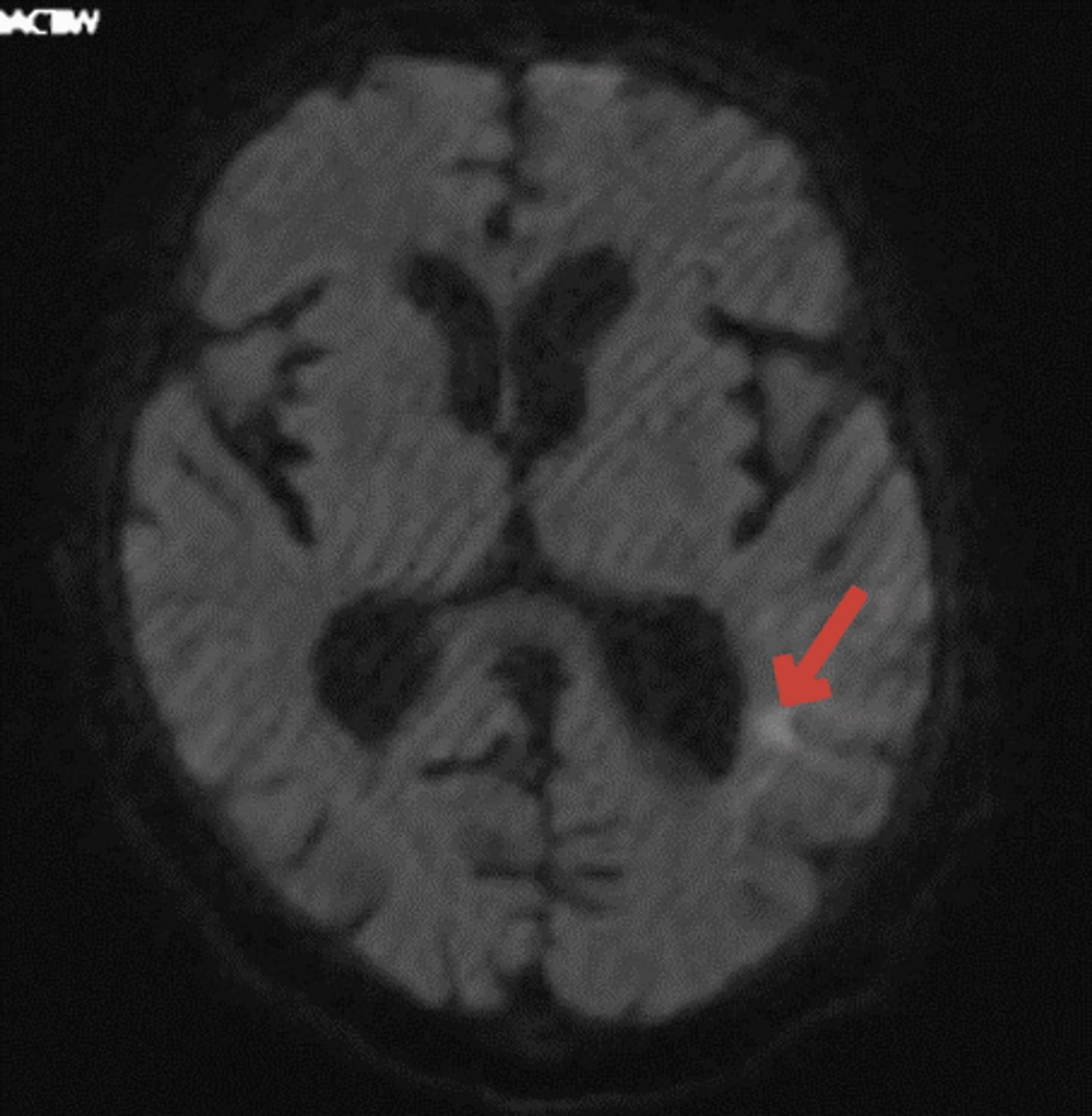
Axial diffusion-weighted MRI of the brain. The image shows high signal in the left temporal lobe on DWI. DWI: diffusion-weighted imaging; ADC: apparent diffusion coefficient; MRI: magnetic resonance imaging. We interpreted DWI hyperintensity alongside the ADC map and clinical timeline because DWI signal alone may be affected by T2 shine-through.

**Figure 5:**
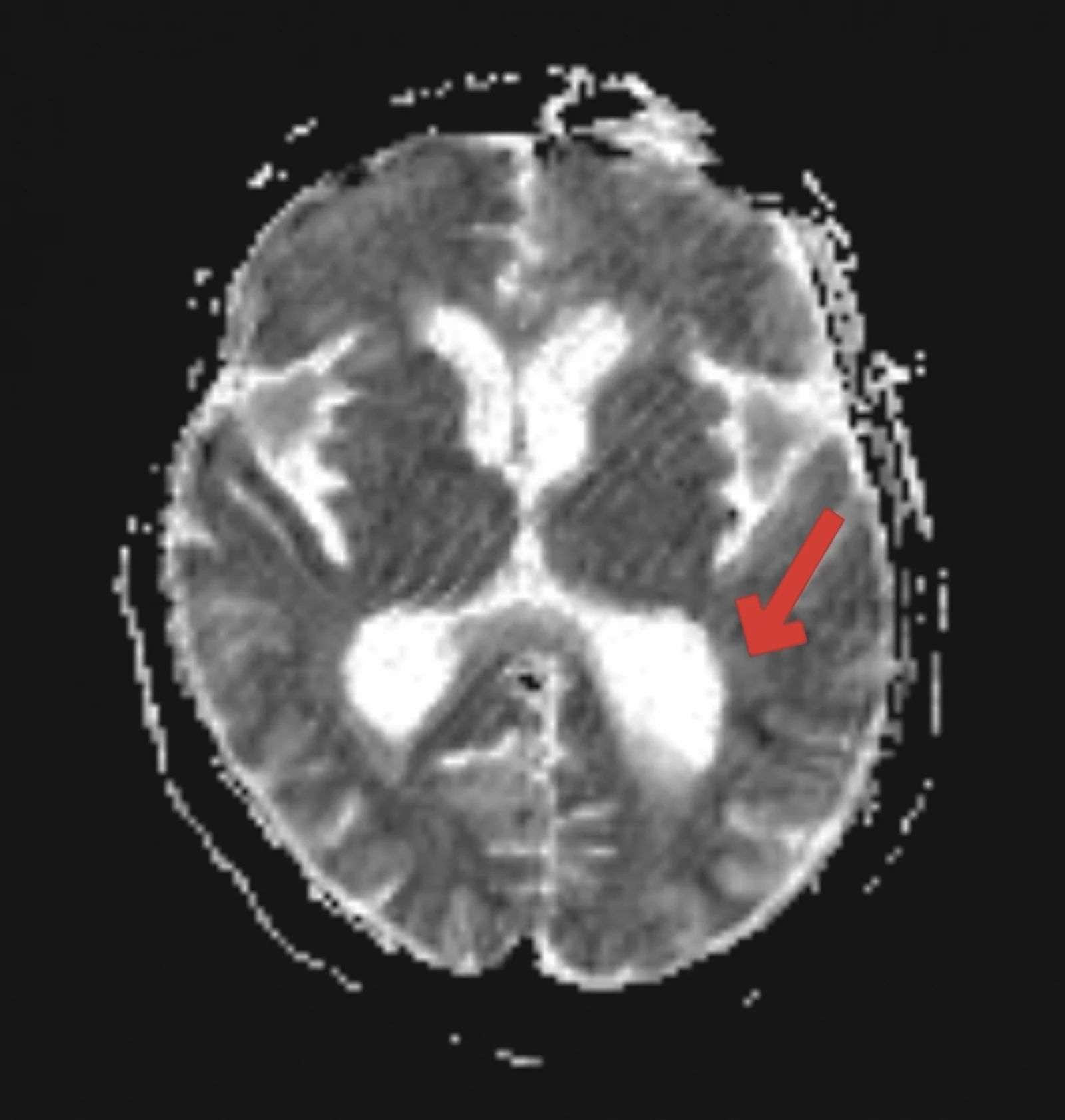
Axial apparent diffusion coefficient map of the brain. The corresponding low-signal focus on the ADC map supports true restricted diffusion when interpreted with the DWI hyperintensity. ADC: apparent diffusion coefficient; DWI: diffusion-weighted imaging. The combined pattern supports recent ischaemic injury, but precise dating depends on the clinical timeline and is limited by movement artefact.

The CHA2DS2-VASc score was 7 [13], and the ORBIT bleeding risk score was 2 [14]. Apixaban was initiated for atrial fibrillation-related thromboembolic prevention, with clopidogrel continued for three weeks before transitioning to apixaban monotherapy. Following identification of Wernicke-type aphasia and an acute left temporal infarct within the left parietotemporal region, presumed to be cardioembolic, management focused on stroke rehabilitation and secondary prevention. Physiotherapy and occupational therapy assessments confirmed no significant motor deficits; the primary functional impairment was language-based. A formal Mental Capacity Act assessment determined that the patient lacked decision-making capacity regarding care and hospital stay, and best-interest decisions were made collaboratively with her family. Discharge planning included stroke rehabilitation and follow-up for aphasia, cognitive function, and heart failure management.

## Discussion

An 83-year-old woman presented with new-onset atrial fibrillation, ischaemic stroke, and Wernicke-type aphasia. Previous studies have reported a high prevalence of atrial fibrillation in patients experiencing a first-ever ischaemic stroke, particularly among women aged 80 years and older [10]. The primary diagnostic challenge was the clinical overlap between delirium and Wernicke-type aphasia in older adults with acute neurological deterioration. The key distinguishing feature, which we did not immediately recognise, was the consistency of the language deficit. In delirium, verbal output and comprehension fluctuate, and may improve with reorientation. In contrast, Wernicke-type aphasia is characterised by fluent jargonistic output, profound comprehension failure, and lack of awareness of the deficit, which remain stable across assessments and reflect a fixed cortical lesion rather than a global attentional disturbance [3,8]. This case received standard delirium management, including reorientation, family support, preservation of the sleep-wake cycle, and avoidance of precipitants such as medications and electrolyte disturbances [15]. However, these approaches do not address aphasia, and the initial focus on delirium delayed stroke-specific assessment and aphasia-focused rehabilitation.

Stroke involving the inferior division of the dominant middle cerebral artery (MCA) may present as fluent Wernicke-type aphasia without hemiparesis, which is a recognised diagnostic pitfall that can delay stroke recognition [16]. In this patient, acute confusion without a clear focal motor deficit initially led to a provisional diagnosis of delirium. However, the persistent pattern of fluent but meaningless speech with profound impairment of comprehension raised suspicion for a focal language disorder. Computed tomography (CT) of the brain identified a left parietotemporal infarct. Given the recent onset of symptoms, magnetic resonance imaging (MRI) was subsequently performed to further characterise the lesion and confirmed acute restricted diffusion in the left temporal lobe. The CT and MRI findings were anatomically compatible, although exact correspondence could not be established due to movement artefact on MRI. The dominant temporal/parietotemporal location and language phenotype were consistent with an inferior-division left MCA distribution, although no MCA thrombus or arterial occlusion was demonstrated.

Marked troponin elevation was observed alongside newly diagnosed atrial fibrillation (AF) and severe heart failure with reduced ejection fraction (HFrEF). Although the patient was initially treated for possible acute coronary syndrome (ACS), cardiology later considered ACS unlikely. According to the Fourth Universal Definition of Myocardial Infarction, a diagnosis requires evidence of acute myocardial ischaemia in addition to an abnormal cardiac troponin pattern [17]. The available record therefore supports myocardial injury associated with haemodynamic stress in a complex cardio-cerebrovascular presentation, rather than a confirmed type 2 myocardial infarction or plaque-rupture ACS. Neuroimaging played a critical role in clarifying the diagnosis. A lesion that is hyperintense on diffusion-weighted imaging (DWI) and hypointense on the corresponding apparent diffusion coefficient (ADC) map supports true restricted diffusion due to cytotoxic oedema and distinguishes it from T2 shine-through, which appears bright on both sequences. However, DWI-ADC appearances alone cannot precisely date an infarct, as ADC values may pseudonormalise during the subacute period [18]. In this case, CT head demonstrated a left parietotemporal infarct. MRI was subsequently performed and showed left temporal DWI hyperintensity with corresponding ADC hypointensity, confirming acute restricted diffusion. The CT and MRI abnormalities were anatomically compatible, although movement artefact prevented exact lesion co-registration. Considering symptom onset approximately 48 hours before admission, persistent new aphasia, and absence of a prior focal language deficit, the MRI findings supported an acute left temporal infarct. Recovery from post-stroke aphasia is influenced by baseline severity, time from stroke, and access to speech and language therapy, underscoring the importance of early and accurate diagnosis for rehabilitation planning [19]. Structured assessment of language comprehension, naming, repetition, reading, writing, and instruction-following provides essential information for distinguishing delirium from aphasia at the bedside. Omission of this assessment was the principal cause of diagnostic delay in this case.

Limitations

This report has several limitations. As a single case study, the findings are not generalisable and do not establish how often aphasia is misattributed to delirium in routine clinical practice. The initial delirium diagnosis relied on a screening assessment and clinical impression; severe aphasia may have affected tasks requiring attention, orientation, and verbal responses, and no comprehensive criterion-based delirium assessment was documented. Aphasia classification was based on detailed bedside assessment rather than a standardised instrument such as the Western Aphasia Battery; thus the description is appropriately restricted to a Wernicke-type profile. Baseline cognition was incompletely characterised because movement artefact affected the MRI. Movement artefact significantly compromised MRI quality, limiting precise lesion delineation. However, CT imaging demonstrated a left parietotemporal infarct. MRI, performed to further characterise the lesion in light of the recent clinical presentation, revealed left temporal DWI-ADC restriction, which, together with the 48-hour clinical timeline, supported an acute left temporal infarct. Movement artefact precluded exact lesion co-registration. Neither CT nor MR angiography demonstrated middle cerebral artery (MCA) occlusion; therefore, the MCA distribution is inferred anatomically. New-onset atrial fibrillation supports presumed cardioembolism, but the record does not include a complete assessment for competing aetiologies. We reconstructed the clinical timeline retrospectively from documentation and collateral history. Despite these limitations, the convergence of the persistent language phenotype, the acute left temporal MRI finding within the left parietotemporal region, and the clinical timeline supports the diagnostic pathway described.

## Conclusions

This case illustrates that Wernicke-type aphasia from an acute left temporal infarct within an anatomically inferred inferior-division left middle cerebral artery territory can be clinically indistinguishable from delirium without formal language assessment. The presence of new-onset atrial fibrillation with elevated cardiac troponin further complicates diagnosis by potentially shifting clinical focus toward a cardiac aetiology. Comprehensive neurological evaluation, emphasising the consistency, fluency, comprehension, and linguistic content of verbal output, is critical for accurate differentiation. CT head revealed a left parietotemporal infarct. Given the recent clinical presentation, MRI was performed for further lesion characterisation and showed left temporal diffusion-weighted imaging hyperintensity with corresponding apparent diffusion coefficient hypointensity, confirming acute restricted diffusion in the left temporal lobe. The MRI findings were anatomically consistent with the left temporal component observed on CT, although movement artefact precluded precise lesion co-registration. Absence of arterial occlusion indicates that the inferior-division left MCA territory remains anatomically inferred, and the presumed cardioembolic mechanism is not definitively established. Troponin elevation in the setting of new AF and severe ventricular dysfunction may reflect secondary myocardial injury rather than an acute coronary syndrome and should not preclude evaluation for a concurrent cerebrovascular event. Multidisciplinary assessment by neurology, geriatrics, cardiology, and speech and language therapy is essential when acute confusion and overlapping cardiac and cerebrovascular findings occur in older adults.

## References

[1] Wilson JE, Mart MF, Cunningham C (2020). Delirium. Nat Rev Dis Primers.

[2] Geriatric Medicine Research Collaborative (2023). Increasing frailty is associated with higher prevalence and reduced recognition of delirium in older hospitalised inpatients: results of a multi-centre study. Eur Geriatr Med.

[3] Marcantonio ER (2017). Delirium in hospitalized older adults. N Engl J Med.

[4] Goldberg TE, Chen C, Wang Y (2020). Association of delirium with long-term cognitive decline: a meta-analysis. JAMA Neurol.

[5] Chung CP (2017). Types of stroke and their differential diagnosis. In: Primer on Cerebrovascular Diseases.

[6] Boehme AK, Martin-Schild S, Marshall RS, Lazar RM (2016). Effect of aphasia on acute stroke outcomes. Neurology.

[7] Engelter ST, Gostynski M, Papa S (2006). Epidemiology of aphasia attributable to first ischemic stroke: incidence, severity, fluency, etiology, and thrombolysis. Stroke.

[8] Lui F, Wroten M (2026). Wernicke Aphasia. https://www.ncbi.nlm.nih.gov/books/NBK441951/.

[9] Li TT, Zhang PP, Zhang MC (2024). Meta-analysis and systematic review of the relationship between sex and the risk or incidence of poststroke aphasia and its types. BMC Geriatr.

[10] Marini C, De Santis F, Sacco S, Russo T, Olivieri L, Totaro R, Carolei A (2005). Contribution of atrial fibrillation to incidence and outcome of ischemic stroke: results from a population-based study. Stroke.

[11] Providência R, Barra S, Paiva L (2013). Atrial fibrillation, elevated troponin, ischemic stroke and adverse outcomes: understanding the connection. Clin Res Cardiol.

[12] Scheltens P, Leys D, Barkhof F (1992). Atrophy of medial temporal lobes on MRI in "probable" Alzheimer's disease and normal ageing: diagnostic value and neuropsychological correlates. J Neurol Neurosurg Psychiatry.

[13] Lip GY, Nieuwlaat R, Pisters R, Lane DA, Crijns HJ (2010). Refining clinical risk stratification for predicting stroke and thromboembolism in atrial fibrillation using a novel risk factor-based approach: the euro heart survey on atrial fibrillation. Chest.

[14] O'Brien EC, Simon DN, Thomas LE (2015). The ORBIT bleeding score: a simple bedside score to assess bleeding risk in atrial fibrillation. Eur Heart J.

[15] Yoon IA, Galarneau D, Winslow M, Park J, Mauricio AX, Reade MC, Teodorczuk A (2025). Diagnosis, prevention and management of delirium: spot it, stop it, treat it. BJPsych Advances.

[16] Arboix A, Alió J (2010). Cardioembolic stroke: clinical features, specific cardiac disorders and prognosis. Curr Cardiol Rev.

[17] Thygesen K, Alpert JS, Jaffe AS, Chaitman BR, Bax JJ, Morrow DA, White HD (2018). Fourth universal definition of myocardial infarction (2018). Circulation.

[18] Srivastava AK, Mehrotra G, Bhargava SK, Agarwal S, Tripathi RP (2008). Studies on the time course of apparent diffusion coefficient and signal intensities on T2- and diffusion-weighted MR Imaging in acute cerebral ischemic stroke. J Med Phys.

[19] RELEASE Collaborators (2021). Predictors of poststroke aphasia recovery: a systematic review-informed individual participant data meta-analysis. Stroke.

